# Dual-domain adversarial learning and feature constraint for unsupervised anomaly detection in chest X-rays

**DOI:** 10.3389/fmed.2026.1757295

**Published:** 2026-07-31

**Authors:** Na Liang, Yehong Tong, Xing Zhang, Xing Wu, Chengliang Wang, Peng Wang

**Affiliations:** 1 College of Computer Science, Chongqing University, Chongqing, China; 2 Chongqing Traditional Chinese Medicine Hospital, Chongqing, China; 3 Centre for Medical Big Data and Artificial Intelligence, The First Affiliated Hospital (Southwest Hospital) of Army Medical University (Third Military Medical University), Chongqing, China

**Keywords:** anomaly detection, chest X-ray, unsupervised learning, rapid screening of pulmonary abnormalities, feature consistency, adversarial learning

## Abstract

**Background:**

Chest X-ray is the standard screening tool for pulmonary diseases, enabling early detection and timely intervention of lung lesions. Due to scarce and costly abnormal labeling of chest X-rays, unsupervised anomaly detection trained only with normal images has become a major research focus, especially, reconstruction-based methods are favored for learning normal data patterns and making it easier to visualize abnormal regions. Although the current reconstruction-based methods of chest X-rays have achieved good result, they have three problems. Firstly, over-reliance on pixel-level alignment in the spatial domain hinders the capture of global information. Secondly, single-layer feature comparison between original and reconstructed images lacks multi-layer and multi-image analysis, resulting in poor feature consistency. Thirdly, using reconstruction error as anomaly score struggles to balance sensitivity to anomalies and fidelity to normal. Using a reconstruction-based approach trained only on normal images, this study improves rapid screening of thoracic abnormalities and shifts screening criteria toward earlier detection of chest lesions.

**Methods:**

We propose an architecture DualA-AD including Dual-domain Adversarial Learning (DAL), Feature Constraint Module (FCM) and Distributed Anomaly Score (DAS). In the training stage, DAL captures image details and global information by integrating spatial and frequency domains for dual-domain adversarial reconstruction. While FCM improves feature consistency between reconstructed and original image by analyzing multi-layer feature difference within individual images and similarity differences across multiple images. In the testing stage, DAS enhances image discriminability by fitting a discriminator’s output on the training set as the real distribution and quantifying the deviation of images from this distribution. On three mainstream public chest X-ray datasets, the model was trained using only normal chest X-rays and evaluated on their respective test sets.

**Result:**

In the experiments on three datasets, DualA-AD achieved the best AUC, ACC, and F1 scores against classical, general, and specialized baselines, with AUC improvements over the SOTA of 0.93, 0.77, and 0.48%, respectively.

**Conclusion:**

DualA-AD improves the accuracy for distinguishing normal and abnormal chest X-rays. The method provides an efficient scheme for fast, low-cost intelligent detection of lung abnormalities, with great potential for computer-aided chest radiographic diagnosis and early prevention and management of pulmonary disorders.

## 1 Introduction

Pulmonary Diseases, including pneumonia, chronic obstructive pulmonary disease (COPD), and the COVID-19 outbreak in 2020, represent a major global health challenge with high morbidity and mortality rates, affecting hundreds of millions of people each year (1). Chest X-ray (CXR) is the most widely used imaging modality for initial screening, diagnosis, and follow-up of pulmonary diseases due to its low cost (2), rapid examination process, and low radiation dose. In clinical practice, training a qualified and experienced radiologist requires a long period, often taking several years to develop proficiency in identifying abnormal shadows such as infiltrates, nodules, consolidations, and pleural effusions. Meanwhile, frontline clinicians, particularly grassroots medical workers, face an overwhelming workload of image interpretation. Most of their clinical time and energy is consumed by a high volume of normal negative cases. Accordingly, leveraging artificial intelligence to shorten preliminary reading time and enhance early screening efficiency has emerged as a vital field at the intersection of medicine and AI. Traditional supervised learning relies heavily on large amounts of annotated “normal” and “abnormal” data. However, in medical imaging, obtaining comprehensive and high-quality annotated data is challenging and costly (3–6), particularly for rare diseases, emerging diseases (such as sudden infectious outbreaks), and complex cases with atypical manifestations. This study employs an unsupervised algorithm trained exclusively on a large number of “normal” (healthy) chest X-rays, enabling the model to learn the representations of healthy lung tissue. Any image that deviates from this “normal pattern” is identified as anomalous. This approach effectively avoids reliance on scarce, unpredictable, and highly variable medical anomaly samples, making it suitable for rapid binary classification (normal vs. abnormal) screening in high-throughput scenarios.

Unsupervised anomaly detection (UAD) methods in chest X-rays, require only normal images for training, play a vital role in lung screening, especially in rare disease recognition. Current UAD methods develop into self-supervised learning (SSL) methods (7) and reconstruction methods (8). SSL methods depend on pretext task designed for specific task to mine supervision signals, which may inadvertently instill biases. In contrast, reconstruction methods train models to reconstruct normal patterns, with using reconstruction error as anomaly score to detect anomalies, have become the mainstream solution in this field.

However, reconstruction methods face three problems: (1) Dependence in spatial domain: they rely on pixel alignment that reflect local information in the spatial domain, ignoring the capture of global information. (2) Absence of feature relation- ship: they only measure single-layer difference between individual original and its reconstruction, ignoring multi-layer feature difference within single images and feature relation among multiple images. (3) Defect of abnormal scores: they use reconstruction error as anomaly score, which does not adapt to the generalization ability of the autoencoder, reducing the difference of anomaly score between normal and abnormal images.

In the paper, based on masked autoencoder (MAE) (9) which is a simple and powerful technology that has been used in anomaly detection (10–12), we propose DualA-AD to distinguish normal and abnormal chest X-ray images. It mines image details and global frequency information through dual- domain adversarial learning and includes a feature constraint module to enhance features consistency between original and reconstructed images. Additionally, a new anomaly score is constructed to separate abnormal and normal images better. Our contributions are as follows:

- We propose Dual-domain Adversarial Learning (DAL) to integrate spatial domain and frequency domain. Pixel discriminator is designed to capture the local information and frequency discriminator is designed to capture the global information, enhancing the ability to learn the normal distribution.
- We propose Feature Constraint Module (FCM) to enhance feature alignment via single to multiple image learning. This module optimizes the multi-layer feature consistency between each image and its reconstruction as well as the global feature consistency across multiple images, which effectively alleviates the feature drift of normal samples.
- We propose Distributed Anomaly Score (DAS) to enhance the distinction between normal and abnormal images. Since the pixel discriminator learns the normal data distribution, fitting its outputs on the training set as the real distribution allows quantifying the distance of images to real distribution, enhancing image discriminability.
- Our model outperforms classical, general and specialized methods. Compared with the SOTA method, the AUC was improved by 0.93, 0.77 and 0.48% on ZhangLab (13), Chexpert (14) and VinCXR (15).

## 2 Related work

Early UAD (16, 17) use hyperplanes to separate normal and abnormal data, but these methods often performed poorly on high-dimensional data. Current UAD in chest X-rays is mainly divided into self-supervised learning (SSL) methods and reconstruction methods.

### 2.1 Self-supervised learning methods

SSL methods rely on pretext tasks designed for specific tasks to extract supervision signals. The pretext tasks include two directions: contrastive learning (18, 41) and pseudo-anomaly generation (19).

Contrastive learning maximizes the similarity between positive pairs and minimizes the similarity between negative pairs to learn data augmentation invariance. Kim et al. (20) used image patches generated from the same lung region as positive pairs and image patches generated from different lung regions as negative pairs, improving the model’s ability to distinguish diseased from healthy areas. Gholamipoor et al. (21) maximized similarity by taking differently augmented views of the same chest image as positive pairs to keep semantic invariance and borrowed MOCO18 to maintain a dynamic queue storing historical features to construct negative pairs.

Pseudo-anomaly generation cuts and pastes on normal samples to artificially create anomaly samples that simulate real exceptions and feed them into the model training classifier along with normal images. Tian et al. (42) simulated abnormal tissue by cutting and pasting small patch regions with visually deformed processed by color jitter, Gaussian noise, and nonlinear intensity transformation from normal images to drive the model to learn abnormal features in local appearance, structure, texture, and color. Sato et al. (7), based on Cutpaste (19), proposed AnatPaste, which randomly selects a region within binary lung lobe regions to draw Gaussian blur elliptical or rectangles and then randomly crops another region and multiplies it with the drawn patch to simulate actual abnormal image.

### 2.2 Reconstruction methods

Reconstruction methods train models on normal images to learn the normal distribution, so during testing, normal images can be effectively reconstructed, while abnormal images lead to significant differences between original and reconstructed image in the reconstruction process. They are divided into two directions: model improvement and anomaly score improvement.

In model improvement, some studies suggest that high residuals in normal regions cause false positives. Mao et al. (8) not only reconstructed input images but also predicted pixel-level reconstruction uncertainty, so errors in normal regions are assigned high uncertainty and thus suppressed, while errors in abnormal regions are assigned low uncertainty and thus highlighted. Bercea et al. (22) refined the reconstruction by estimating the dense deformation field, aligning the reconstruction with the input image and reducing the residual error in normal regions, thereby significantly reducing false positives in normal images. Some studies explored the memory capability of models for normal images, hindering the reconstruction of abnormal images during testing. Xiang et al. (22, 24) proposed a perceptual memory bank behind the encoder to store normal patterns using the consistent characteristics of chest X-rays, so during testing, the normal patterns closest to the test images are selected from the memory bank for reconstruction and the reconstructed image theoretically does not contain defects, thus increasing reconstruction errors for abnormal images. Tian et al. (25) applied self-attention to the encoder to memorize normal patterns, leveraging multi- level memory information as prior features, and reconstructed missing normal pixels via cross-attention, preventing accurate anomaly reconstruction. However, the existing methods depend on spatial domain and do not achieve feature alignment from multiple angles, we propose dual domain adversarial learning and feature constraint module.

In anomaly score improvement, Sota et al. (26) encoded both original and reconstructed images into multi-layer latent variables, capturing the hierarchical feature differences from the global structure to the local details as the anomaly score, thus enabling a more comprehensive evaluation of anomalies. Bhatt et al. (27) calculated anomaly score by combining the deviations of the original and reconstructed images in image space, latent space, and discriminator features, considering multi-level image information to captured anomalies more comprehensively, especially low-contrast nodules. However, the error between original and reconstructed images cannot guarantee image discrimination, so we propose distributed anomaly score.

## 3 Method

### 3.1 Overview

**Figure 1:**
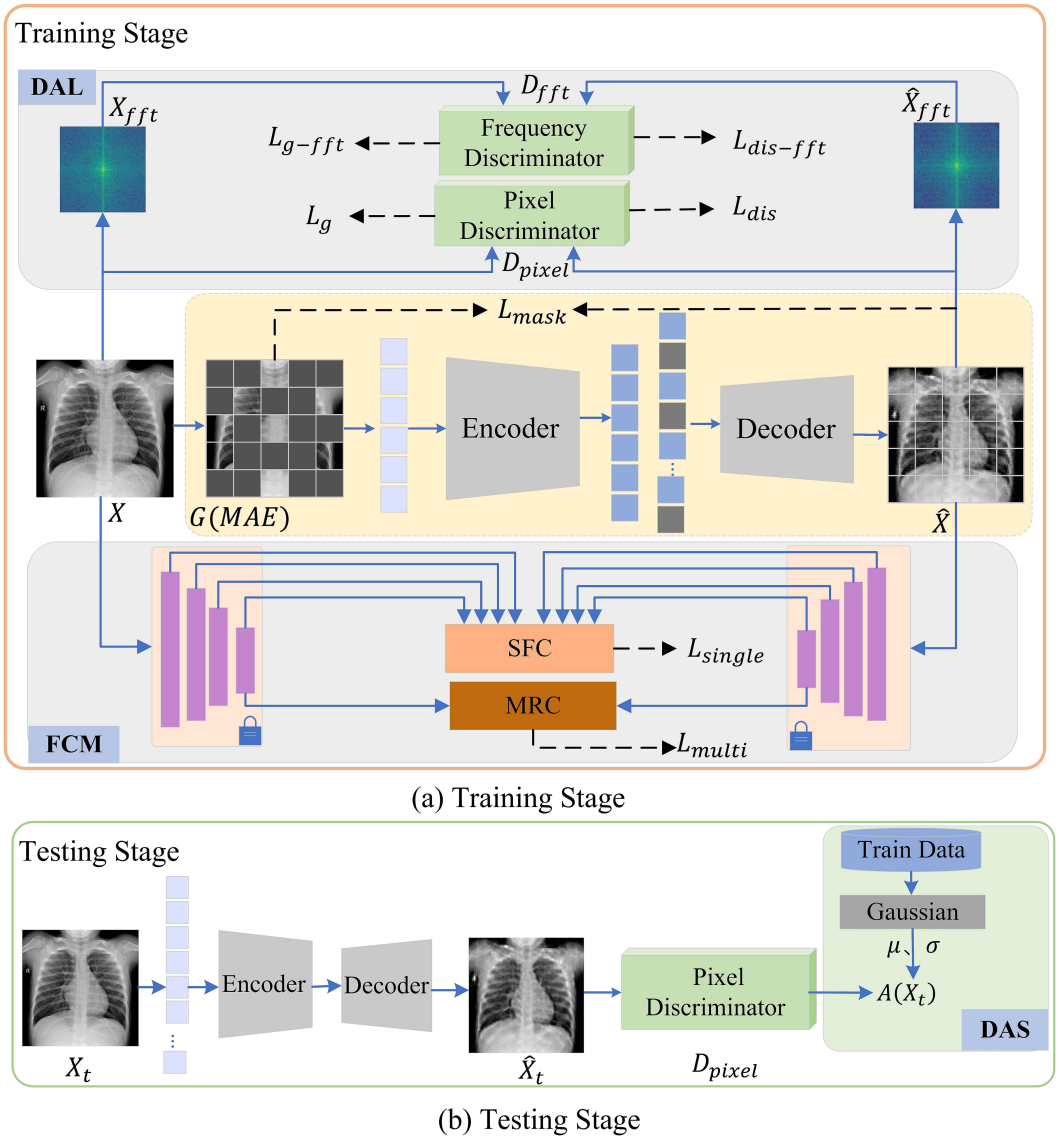
The architecture of DualA-AD. **(a)** Is training stage including MAE reconstruction network with DAL and FCM. **(b)** Is testing stage including DAS.

The architecture of DualA-AD is shown in Figure 1. It consists of MAE reconstruction network, Dual-domain Adversarial Learning (DAL) and Feature Constraint Module (FCM) in the training stage and Distributed Anomaly Score (DAS) in the testing stage. In Figure 1a, using MAE as generator *G*, DAL employs pixel adversarial learning and frequency adversarial learning to enhance reconstruction details and capture image global information. FCM computes multi-layer differences within single image while also calculating similarity differences across multiple images. In Figure 1b, DAS uses Gaussian function to construct new anomaly score from the pixel discriminator and quantify the gap between test image and real distribution, improving the discrimination of the image. Yellow part in Figure 1a, the input normal image X is divided into patches, with 75% randomly masked. The remaining 25% of patches are fed into the encoder and the decoder reconstructs the image *X̂*. The loss $L_{\text{mask}}$ is calculated pixel by pixel using MSE for masked patches $L_{\text{mask}=}\text{mask}{\left\|X-\hat{X}\right\|}^{2}$ , where “mask” is a weight of 1 to masked patches and 0 to visible patches.

### 3.2 Dual-domain adversarial learning

To address the dependence on the spatial domain while ignoring information from other domain, we propose DAL to integrate the spatial domain with local information and the frequency domain with global information. That way, the model can capture local and global information, improving reconstruction accuracy under adversarial strategy.

#### 3.2.1 Pixel discriminator

To refine the understanding at the pixel level and capture image details, the pixel discriminator $D_{\text{pixel}}$ is designed to learn the normal distribution in the way of game. Specifically, $D_{\text{pixel}}$ consists of five convolutional blocks (each with a 5 *×* 5 kernel, stride 2, BatchNorm2d and ReLU) followed by a fully connected regressor that maps flattened features to target values. $D_{\text{pixel}}$ judges the original image X and reconstructed images $\hat{X}$ as real or fake, with real label as 1 and fake label as 0. During this period, two types of losses are incurred: adversarial loss and discriminator loss. Adversarial loss *L_g_* indicates how close the reconstructed image is to the normal distribution. *X̂* is fed into $D_{\text{pixel}}$ to get the output and the difference between the output and the real label is measured to compute $\;L_{g}$ acting on *G*. By minimizing $\;L_{g}$ , *G* is optimized to generate reconstructed images that are closer to the real image as given in Equation 1.

$$\min\;L_{g}=\log(1-D_{\text{pixel}}(G(X)))$$ (1)

Discriminator loss $\;L_{\mathrm{dis}}$ indicates the ability of $D_{\text{pixel}}$ to distinguish between the original image and the reconstructed image. The loss acts on $D_{\text{pixel}}$ when $\;D_{\text{pixel}}$ discriminates reconstructed image as fake and original image as real. In this process, $D_{\text{pixel}}$ and *G* form an adversarial game, where *G* generates reconstructed images that closely match the normal distribution, so that $D_{\text{pixel}}$ cannot distinguish the recon- structed image from the original image, effectively learning the normal distribution. Therefore, maximizing $L_{\mathrm{dis}}$ to drives this competitive process (as given in Equation 2).

$$\max\;L_{\mathrm{dis}}=\log(D_{\text{pixel}}(G(X))+\log(1-D_{\text{pixel}}(X))$$ (2)

#### 3.2.2 Frequency discriminator

Frequency domain analysis preserves the overall information of image more effectively than spatial domain. Xie et al. (28) and Ni et al. (29) used high or low frequency filters to mask input images, improving frequency domain perception. But this method is unsuitable for masked reconstruction, as high masking in the spatial and frequency domains causes significant information loss of visible patch, weakening image recovery and feature extraction. In chest X-rays, high frequency information primarily represents textures or sharp area and low frequency information represents the anatomical structure. To build a model that captures global information across all frequencies, we design a frequency discriminator $D_{\mathrm{fft}}$ that takes the frequency representation of X and $\hat{X}$ as input.

Specifically, we use single-channel representation of X and $\hat{X}$ to focus on the overall brightness characteristics of image and then frequency representation $X_{\mathrm{fft}}$ and ${\hat{X}}_{\mathrm{fft}}$ are obtained by 2D Discrete Fourier Transform F(x) (as given in Equation 3):

$$F(x)(u,v)=\sum_{h=0}^{H-1}\sum_{w=0}^{W-1}f(h,w)e^{-i2\pi (\frac{\mathrm{uh}}{H}+\frac{\mathrm{vw}}{W})}$$ (3)

where the single-channel image size is *H* × *W*. f(h,w) is the pixel value at the spectral coordinates (h,w) . F(x)(u,v) the complex frequency value at the spectral coordinates (*u*, *v*), with e being the Eulerian number and *i* being the imaginary unit.

Frequency representation $X_{\mathrm{fft}}$ and ${\hat{X}}_{\mathrm{fft}}$ are fed into the $D_{\mathrm{fft}}$ which works on the same principle as $D_{\text{pixel}}$ . The real label is 1 and the fake label is 0. Minimizing the frequency adversarial loss $L_{g-\mathrm{fft}}$ fed back to *G* (as given in Equation 4):

$$\min\;L_{g-\mathrm{fft}}=\log(1-D_{\mathrm{fft}}(F(\hat{X})))$$ (4)

Maximizing the frequency discriminator loss $L_{\mathrm{dis}-\mathrm{fft}}$ fed back to $D_{\mathrm{fft}}$ (as given in Equation 5):

$$\max\;L_{\mathrm{dis}-\mathrm{fft}}=\log(D_{\mathrm{fft}}(F(X))+\log(1-D_{\mathrm{fft}}(F(\hat{X})))$$ (5)

### 3.3 Feature constraint module

The current single-layer feature difference within single image cannot effectively achieve feature alignment, so we propose FCM including Single-image Multi-layer Feature Consistency (SFC) to indicate the feature gap of each the original and reconstructed images at different levels and Multi-image Feature Relation Consistency (MRC) to measure the overall consistency of the original and reconstructed images.

#### 3.3.1 Single-image multi-layer feature consistency

To evaluate single image feature consistency and emphasize perceptual quality of reconstructed image, we calculate feature difference between original and reconstructed image at different layers.

Specifically, since different layers of the VGG reflect various texture and structural information, we use the official pre-trained VGG-19 to compute the loss between the original and reconstructed image. We extract features from layers{1, 6, 11, 20} which is grounded in convolution kernel size transitions in VGG-19, ensuring that the model can cover image features from the low level to the high level. The VGG weights are frozen during training to avoid interference with model training, ensuring the stability of feature extraction.

**Figure 2:**
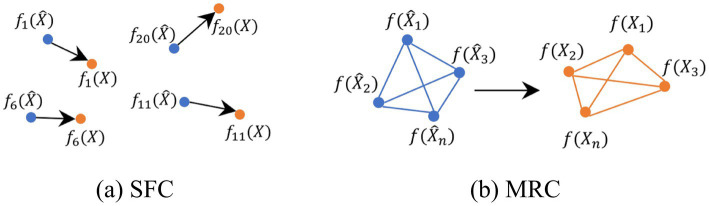
The calculation process of feature consistency, **(a)** is Single-image Multi-layer Feature Consistency (SFC), **(b)** is Multi-image Feature Relation Consistency (MRC).

In Figure 2a, $f_{k}(X)$ and $f_{k}(\hat{X})$ represent the features of original image and corresponding reconstructed image at the *k*-th layer of VGG network, respectively. The feature differences between $f_{k}(X)$ and $f_{k}(\hat{X})$ in different layers are minimized to enhance the consistency within single image in deep feature space. So $L_{\text{Single}}$ is defined as Equation 6:

$$\;L_{\text{Single}}=\alpha \sum_{k}{\left(f_{k}(X)-f_{k}(\hat{X})\right)}^{2}$$ (6)

where α is loss weight, taken as 0.05 based on experience and *k* ∈ {1, 6, 11, 20}.

#### 3.3.2 Multi-image feature relation consistency

Liu et al. (30) introduce pair distillation by computing pixel-wise similarity matrices within a teacher output, as well as student output, enforcing consistency through the differences of two similarity matrices. Inspired by this, we propose MRC to estimate the discrepancy between the pairwise similarity of reconstructed images and that of original images in each batch, ensuring the distribution of reconstructed images aligns with original images and enhancing overall consistency.

In Figure 2b, assuming a batch size of n, for the batch of original images $X_{i}$ and reconstructed images ${\hat{X}}_{i}$ , we pass them through all the convolutional layers of the pre-trained VGG-19 network and obtain two sets of feature maps $F_{B}=\{f(X_{1}),f(X_{2}),\cdots ,f(X_{n})\}$ and ${\hat{F}}_{B}=\{f({\hat{X}}_{1}),f({\hat{X}}_{2}),\cdots ,f({\hat{X}}_{n})\}.$ Then the pairwise cosine similarity is computed between the feature maps FB, where aij represents the similarity between $f(X_{i})$ and $\left(X_{j}\right)$ . Similarly, the pairwise cosine similarity is computed between the feature map ${\hat{F}}_{B}$ , where ${\hat{a}}_{\mathrm{ij}}$ represents the similarity between $f({\hat{X}}_{i})$ and $f({\hat{X}}_{j})$ . Finally, the loss $\;L_{\text{multi}}$ is obtained by calculating the squared difference between these pairwise similarities (as given in Equation 7).

$$L_{\text{multi}}=\frac{1}{n\times n}\sum_{i=1}^{n}\sum_{j=1}^{n}{\left(a_{\mathrm{ij}}-{\hat{a}}_{\mathrm{ij}}\right)}^{2}$$ (7)

In our implementation, the cosine similarity between two features $f(X_{i})$ and $f(X_{j})$ is defined as Equation 8:

$$a_{\mathrm{ij}}=\frac{\;f(X_{i})\cdot f(X_{j})}{\;{\left\|f(X_{i})\right\|}_{2}\;{\left\|f(X_{j})\right\|}_{2}}$$ (8)

### 3.4 Distributed anomaly score

For anomaly detection, we employ a discriminator that have learned normal distribution to assess the probability of anomalies, allowing the model to autonomously determine deviation likelihood. Since $D_{\text{pixel}}$ can detect local anomalies more effectively so we construct DAS based on its outputs.

We use Gaussian distribution (31) to fit the real distribution reflected by the training set and calculate the distance from the test images to the real distribution, thus quantifying the degree of deviation of image. The inputted images are zero-masked.

Firstly, we randomly select 20% from the training set, denoted as I′, to calculate the mean *μ* (as given in Equation 9) and standard deviation *σ* (as given in Equation 10), with n′ being size of I′.

$$\mu =\frac{\sum_{X^{\prime }\in I^{\prime }}D_{\text{pixel}}(G(X^{\prime }))}{n^{\prime }}$$ (9)

$$\sigma =\sqrt{\frac{\sum_{X^{\prime }\in I^{\prime }}{\left(D_{\text{pixel}}(G(X^{\prime }))-\mu \right)}^{2}}{n^{\prime }}}$$ (10)

Then the model processes the test image *X_t_* and output v through *D*_*pix*el_ and the difference from test image to real distribution is quantified for anomaly detection to get the final anomaly score $A(X_{t})$ (as given in Equations 11,12).

$$v=D_{\text{pixel}}(G(X_{t}))$$ (11)

$$A(X_{t})=\varphi (\frac{v-\mu }{\sigma })\;\;$$ (12)

where *φ* is sigmoid function, mapping $A(X_{t})$ within [0,1]. When both normal and abnormal images exist, $A(X_{t})$ denotes the probability that the image is anomalous, with a larger value indicating a higher likelihood of anomaly. $A(X_{t})$ closer to the two boundaries (0 for normal samples and 1 for abnormal samples) correspond to higher classification certainty, whereas scores falling in the intermediate range near the threshold signify ambiguous lesions and feature confusion within images, indicating high predictive uncertainty.

## 4 Experimental analysis and results

### 4.1 Experimental setup

#### 4.1.1 Datasets

Our model was tested on three publicly available standard benchmark datasets, called ZhangLab, Chexpert, and VinCXR. All data is processed in an unsupervised manner, that is, only normal images are used during the training stage and the testing stage includes both normal and abnormal images. Table 1 shows the details of the training set and test set.

**Table 1:** The details about the use of three datasets.

Dataset	Train	Test
ZhangLab	1,349 normal	234 normal + 390 abnormal
Chexpert	4,449 normal	250 normal + 250 abnormal
VinCXR	4,000 normal	1,000 normal + 1,000 abnormal

ZhangLab dataset (13) was certified by experts and released by the University of San Diego in 2018. It is officially divided into training set with 1,349 normal and 3,883 abnormal images and test set with 234 normal and 390 abnormal images. The abnormal images only include pneumonia images.

Chexpert dataset (14) was introduced by Stanford University in 2019 and popular in chest X-ray disease identification competitions. It includes a total of 12 different anomalies. Using the partition of the front-view PA images from SQUID (23), the training set contains 4,449 normal images and the test set contains 250 normal and 250 abnormal images (at least 10 images for each abnormal type).

VinCXR dataset (15) was collected primarily from two hospitals in Vietnam and include 14 categories of anomalies in total. Using the partition of DDAD (32), the training set contains 4,000 normal images and the test set contains 1,000 normal and 1,000 abnormal images.

#### 4.1.2 Evaluation mertric

A set of metrics is used to evaluate the performance of the proposed methods, including the Receiver Operating Characteristic (ROC) curve, Area Under the ROC Curve (AUC), Accuracy (ACC) and F1 Score (F1).

The ROC curve is defined by two key metrics: the True Positive Rate (TPR) and the False Positive Rate (FPR), which illustrate the model’s classification performance across all possible decision thresholds. The AUC quantifies the overall classification ability of the model by measuring the area under the ROC curve, serving as a robust indicator of classification performance. Higher values of ACC, F1, and AUC indicate better model performance.

#### 4.1.3 Implementation details

This experiment is implemented using the PyTorch framework and conducted on a single NVIDIA RTX 4090 GPU. The model is trained for 300 epochs using the Adam optimizer, with the base random seed set to 0. For the discriminator, the Adam optimizer is used (first-moment coefficient *β*₁ = 0.5, second-moment coefficient β₂ = 0.999). The MAE architecture adopts ViT-Base-patch16, initialized with official pre-trained weights, and uses the Adam optimizer (β₁ = 0.9, β₂ = 0.95). All training input images are uniformly resized to 224 × 224 (three-channel), with a patch size of 16 × 16 and a mask ratio of 0.75. Data augmentation is applied to the training images, including random rotation ±10°, random translation ±5%, and random scaling 95–105%, followed by standardization using dataset statistics. Test images undergo only resizing and standardization, without any data augmentation. On each dataset, three independent training runs are conducted using the same parameters, and the average performance metrics are reported.

### 4.2 Comparison experiment

To evaluate the effectiveness of DualA-AD, DualA-AD is compared with three types of reconstruction methods: classical methods, general medical methods and specialized methods. For models that released source code and were reproducible, we reproduced the code and conducted experiments. For other models, if the original papers already reported results on the datasets used in this study, we directly extracted those results; otherwise, for the ZhangLab and Chexpert datasets, results were taken from SQUID, and for the VinCXR dataset, results were taken from DDAD.

- For classical methods, we choose Ganomaly (33), f-AnoGAN (34) and MemAE (35). In the field of anomaly detection, these three methods are often used for comparison.
- For general medical methods, we choose methods of conducting experiments using the chest X-ray dataset for comparison, including SALAD (36), EDC (37), Hetero-AE (38) (SOTA for ZhangLab) and D2UE (39) (SOTA for Chexpert and VinCXR). Due to unavailable code and dataset differences, the results of SALAD and Hetero-AE are extracted from the paper.
- For specialized methods designed specifically for chest X-rays, we choose AE-U (8), MorphAEus (19), SQUID (20) and SimSID (21) (SOTA for three datasets). Due to unavailable code and dataset differences, the results of AE-U are extracted from the paper.

We mainly analyzed the AUC value. As demonstrated in Table 2, DualA-AD achieves superior performance across all three datasets. The conclusions are the following:

- DualA-AD is far superior to classical methods. Compared with classical methods, DualA-AD achieves an impressive increase of more than 10% in AUC on three datasets. This significant advantage fully demonstrates the advanced nature of DualA-AD in feature extraction and optimization.
- DualA-AD is significantly superior to general methods. DualA-AD outperforms others on all metrics in the three datasets. It achieves an AUC improvement of 1.81% over Hetero-AE on ZhangLab, while outperforming D2UE by 2.98 and 2.14% on Chexpert and VinCXR, respectively. This indicates that DualA-AD is specifically optimized for the characteristics of chest X-rays, effectively extracting key features related to chest X-rays.
- DualA-AD is superior to specialized methods. Compared with Sim-SID, DualA-AD achieves an AUC improvement of 0.93, 0.77 and 0.48% on three datasets. This indicates the effectiveness of DualA-AD in utilizing dual-domain information and optimizing multi-layer feature consistency and global feature consistency.

**Table 2:** The results (%) of comparisons with other reconstruction method.

Type	Method	Source	ZhangLab			Chexpert			VinCXR		
			AUC	ACC	F1	AUC	ACC	F1	AUC	ACC	F1
Classical	Ganomaly^†^	ACCV’18	78.00	70.00	79	68.90	65.70	65.10	59.60	–	–
	f–AnoGAN^†^	MIA’19	75.50	74.00	81.00	65.80	63.70	59.40	–	–	–
	MemAE^†^	ICCV’19	77.80	56.5	82.6	54.30	55.60	53.30	55.80	–	–
General	SALAD*	TMI’21	82.65	75.92	82.14	–	–	–	–	–	–
	EDC	TMI’23	89.25	83.49	87.2	70.08	64.20	70.02	67.16	60.10	67.80
	D2UE	MICCAI’24	72.08	62.50	63.32	80.15	75.00	74.59	73.37	68.26	68.78
	Hetero–AE*	TIP’24	90.13	84.93	88.22	–	–	–	–	–	–
Specialized	AE–U*	MICCAI’20	78.00	–	75.00	–	–	–	73.80	–	–
	MorphAEus	MICCAI’2	79.42	75.56	79.94	67.12	64.45	56.93	69.97	65.77	64.67
	SQUID	CVPR’23	89.32	84.50	87.79	79.77	74.00	75.29	73.02	67.35	66.04
	SimSID	TPAMI’24	91.01	85.65	88.56	82.36	75.20	76.60	75.03	69.50	69.98
	DualA–AD	Ours	**91.94**	**86.46**	**89.11**	**83.13**	**77.50**	**76.92**	**75.51**	**70.40**	**71.40**

Bold marks best result. *Indicates the results are extracted from the paper, but the result of AE-U on VinCXR is extracted from DDAD. ^†^Indicates results are extracted from SQUID On ZhangLab and Chexpert and results are extracted from DDAD On VinCXR.

### 4.3 ROC curves comparison

We conducted a experiment with several methods including EDC (37), D2UE (39), MorphAEus (19) SQUID (20) and SimSID (21), all of which provide publicly available implementations. The experiment was performed through ROC curve analysis, which illustrates the critical trade-off between sensitivity (True Positive Rate) and specificity (False Positive Rate).

**Figure 3:**
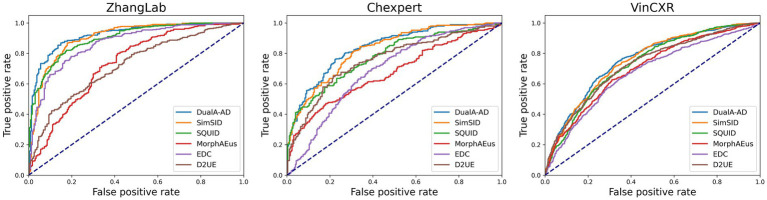
Receiver operating characteristic curve (ROC) of the comparison methods on three datasets.

In Figure 3, the blue curves representing DualA-AD achieve an optimal balance between sensitivity and specificity, demonstrating the effectiveness of DualA-AD in anomaly detection tasks. Particularly in complex scenarios requiring high precision and high recall, DualA-AD can provide more reliable detection performance.

### 4.4 Ablation experiment

**Figure 4:**
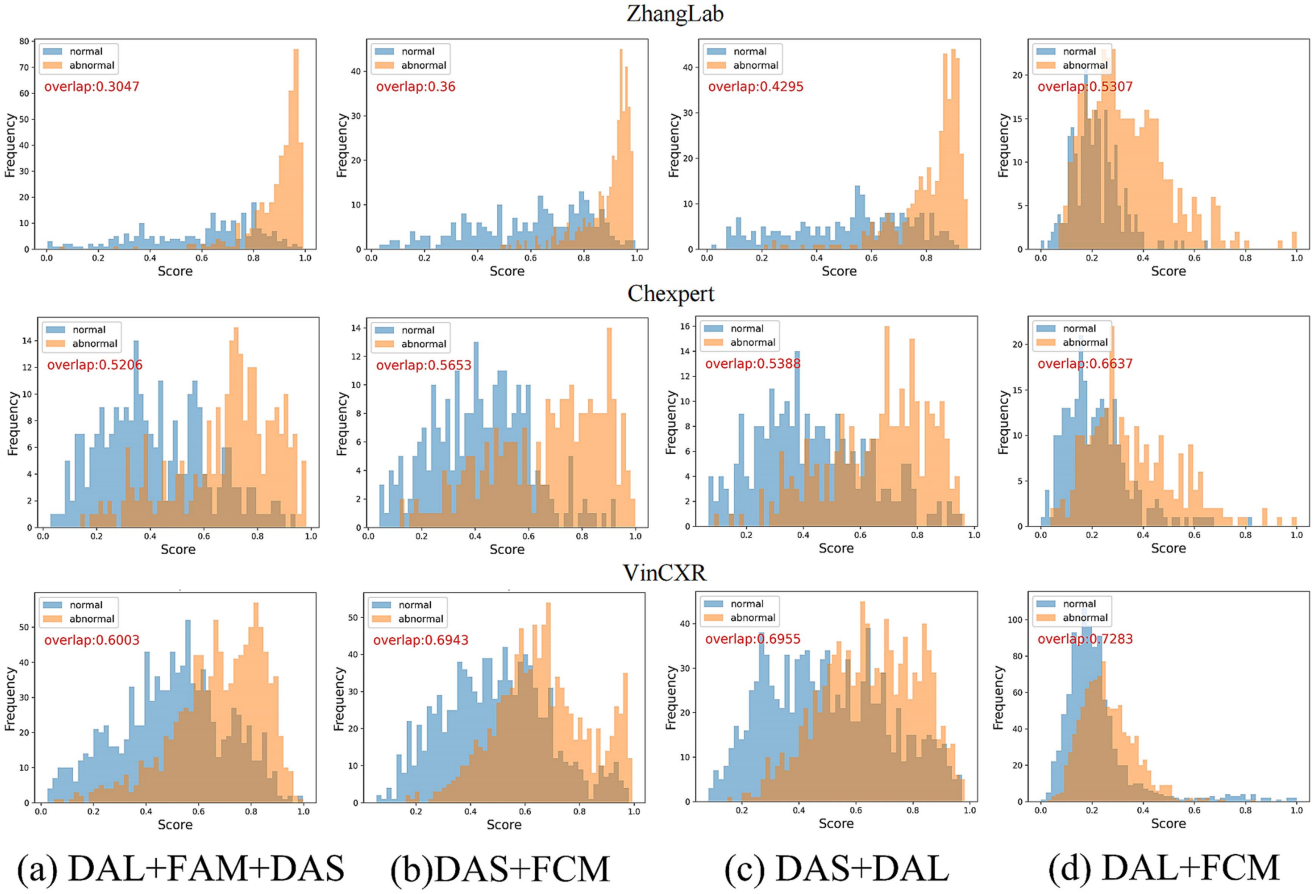
Histogram of anomaly scores for normal (blue) and abnormal (orange) images by adding different components. Red values denote anomaly score overlap; less overlap indicates better detection performance. The horizontal axis shows normalized anomaly scores, and the vertical axis shows the count. **(a)** is the result of DAL+FAM+DAS, **(b)** is the result of DAS+FCM, **(c)** is the result of DAS+DAL, **(d)** is the result of DAL+FCM.

We evaluated the effectiveness of each component on three datasets, with Table 3 and Figure 4 showing the results and anomaly score histograms, respectively. It is important to note that the construction of DAS is derived from the output of $D_{\text{pixel}}$ , therefore $D_{\text{pixel}}$ is indispensable when using DAS in the paper.

**Table 3:** The results (%) of adding different components on three datasets.

DAL		FCM			ZhangLab			Chexpert			VinCXR		
$D_{\text{pixel}}$	$D_{\mathrm{fft}}$	SFC	MRC	DAS	AUC	ACC	F1	AUC	ACC	F1	AUC	ACC	F1
					67.55	60.89	61.39	76.00	70.20	68.79	60.63	58.05	60.14
√					73.73	69.71	74.56	76.75	71.00	72.16	64.13	61.15	58.33
√	√				73.62	70.19	75.78	77.18	73.00	71.09	68.56	65.85	64.22
√				√	83.33	76.12	78.68	77.01	71.80	72.5	67.87	64.4	65.36
√	√			√	89.22	82.05	85.14	79.96	73.60	74.52	70.29	66.65	70.63
√		√		√	89.95	81.89	84.54	80.55	75.60	72.38	71.23	64.90	65.21
√		√	√	√	90.23	83.81	86.03	81.44	75.60	75.79	71.51	66.70	69.56
√	√	√	√	√	91.94	86.46	89.11	83.13	77.50	76.92	75.51	70.40	71.40

Effectiveness of DAS. Based on MAE + $D_{\text{pixel}}$ with reconstruction error as the anomaly score (row 2 of Table 3), the addition of DAS improves the AUC by 9.6, 0.26 and 3.68% on three datasets (row 4 of Table 3). And removing DAS leads to feature overlap increases of 0.226, 0.1431 and 0.128 when comparing Figures 4a,d. It demonstrates that DAS, which models the output distribution of $D_{\text{pixel}}$ , can extract discriminative features beyond reconstruction errors and enlarge the score discrepancy between normal and abnormal samples.

Effectiveness of DAL. The addition of DAL improves the AUC by 6.07, 1.18 and 7.9% in three datasets (row 1 and row 3 of Table 3). This indicates that DAL achieves dual-domain complementarity in both spatial and frequency domains. It calibrates the global intensity distribution, thus addressing the inherent flaw of spatial-only methods that neglect checkerboard artifacts during reconstruction.

DAL consists of $D_{\text{pixel}}$ and $D_{\mathrm{fft}}$ . After introducing the $D_{\text{pixel}}$ , the AUC values on the three datasets rise by 6.81, 0.75 and 5.61%, respectively, (row 1 and row 2 of Table 3). It demonstrates that the pixel discriminator enables fine-grained understanding and captures subtle image details at the pixel level. Additionally, adding $D_{\mathrm{fft}}$ brings extra AUC improvements of 5.89, 2.95 and 2.25% in three datasets (row 4 and row 5 of Table 3). And decreases the overlap by 0.1248, 0.0182 and 0.0952 (Figures 4a,d). This result indicates that $D_{\mathrm{fft}}$ effectively process frequency-domain information and preserve the global contextual information of images.

Effectiveness of FCM. Removing FCM (combined with SFC and MRC) decreases the AUC by 2.72, 3.17 and 5.22% on three datasets (row 4 of Table 3) and increases the overlap by 0.1248, 0.0182 and 0.0952 (Figures 4a,c). Moreover, based on DAS (row 2 of Table 3), the addition of FCM enhances the AUC (row 5 of Table 3). This indicates that FCM suppresses reconstruction distortion, improves global feature coherence and mitigates feature drift. It further regularizes DAL by limiting optimization to a compact feature space, clustering normal sample features and strengthening the separation between normal and abnormal inputs for DAS.

FCM consists of SFC and MRC. After introducing the SFC, the AUC values on the three datasets rise by 6.62, 3.54 and 3.1%, respectively, (row 4 and row 6). This demonstrates that SFC can effectively reduce the feature gap between original images and reconstructed images across multiple network layers. Additionally, adding the MRC brings extra AUC improvements of 0.28, 0.89 and 0.28%, respectively, (row 6 and row 7), which indicates that MRC further enhances global feature consistency and cross-image feature alignment.

### 4.5 Visualization

#### 4.5.1 Visualization of DAL and FCM

**Figure 5:**
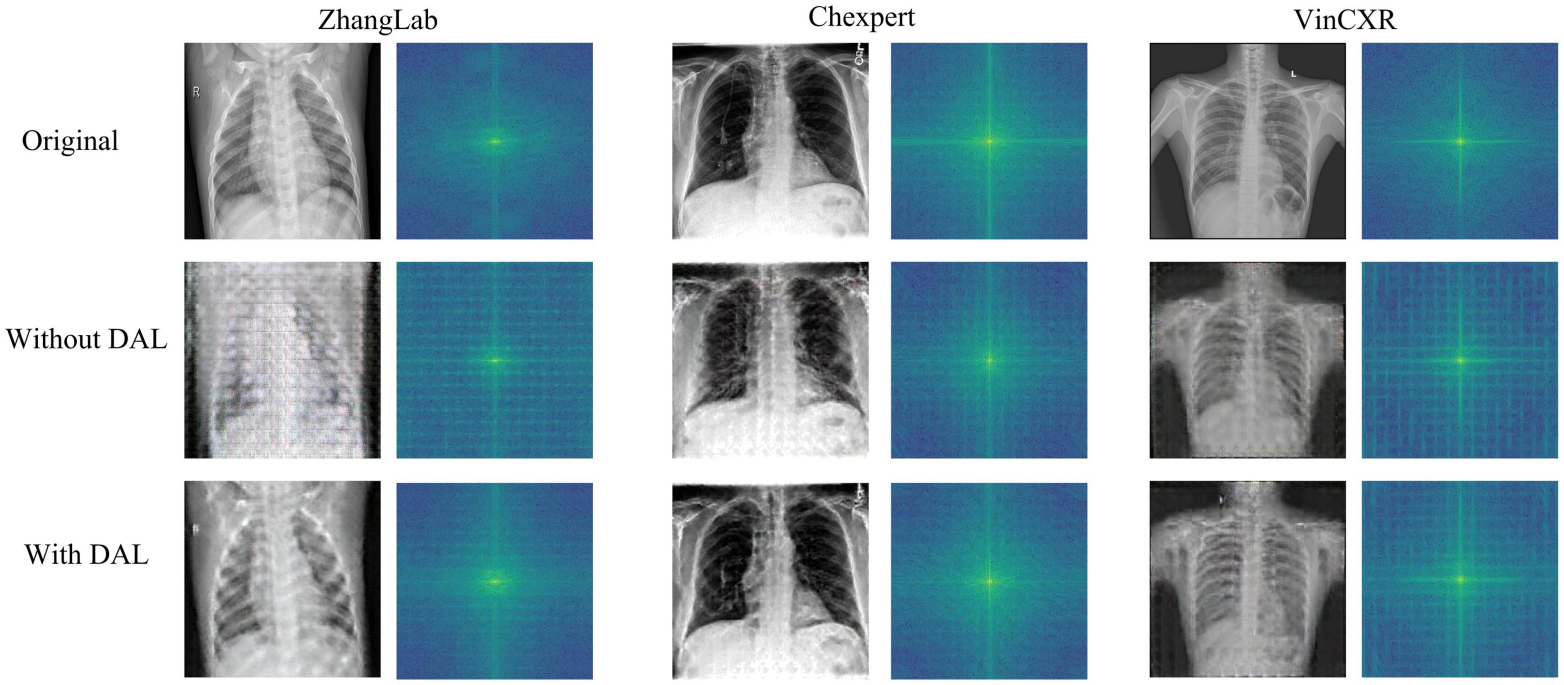
Frequency map of reconstruction before and after adding DAL.

**Figure 6:**
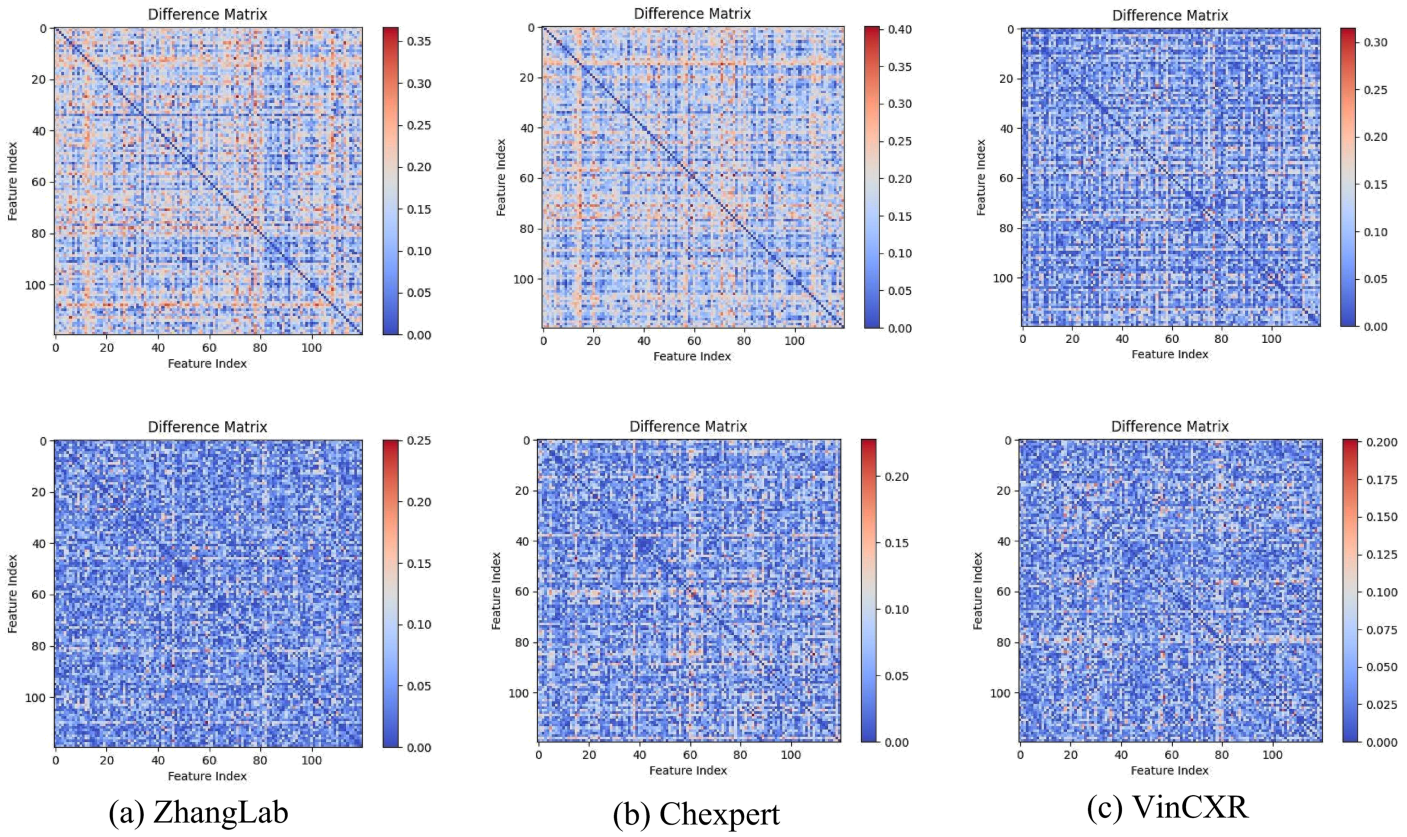
Difference map of relationships before and after adding FCM, the first and second lines represent without FCM and with FCM. **(a)** is the result on ZhangLab dataset, **(b)** is the result on Chexpert dataset, **(c)** is the result on VinCXR dataset.

In Figures 5, 6, we visualized DAL and FCM based on MAE with DAS as the anomaly score. In Figure 5, comparing the third row with the second row, the checkerboard artifacts of the image spectrum (magnified for viewing) are suppressed with DAL, indicating DAL regularizes the frequency spectrum of reconstructed images and the effectiveness in capturing global information. In Figure 6, we compute the relational difference map by subtracting the reconstructed image’s feature similarity from the original image’s feature similarity. It shows that after adding FCM, difference map of feature relation decreases, indicating that FCM narrows the feature drift among normal samples and improve the global consistency.

#### 4.5.2 Distribution validation of DAS

**Figure 7:**
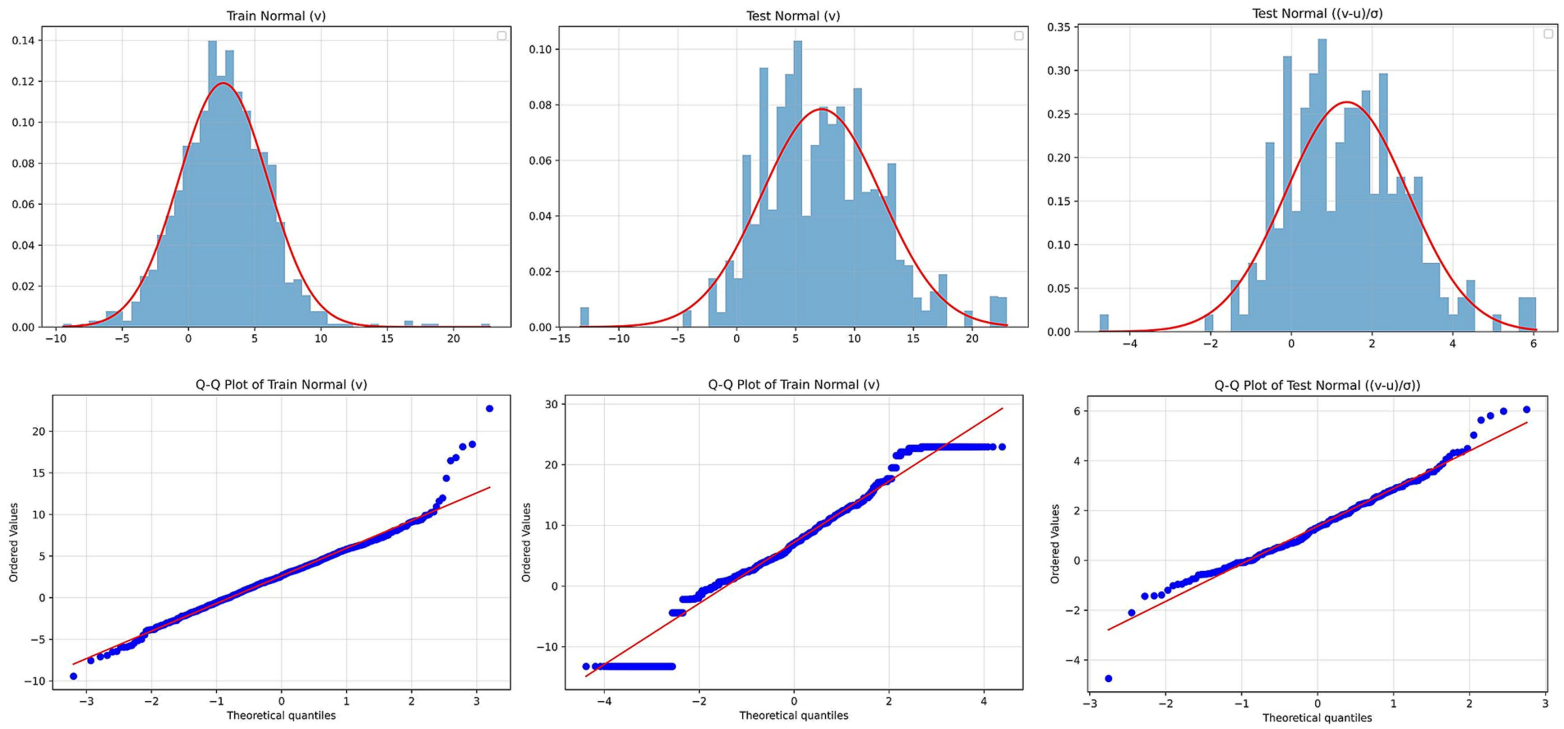
Distribution verification of the pixel discriminator outputs for normal chest X-rays.

To verify the Gaussian assumption adopted in DAS, we visualize the distribution of discriminator outputs via fitted histograms and *Q*–*Q* plots as shown in Figure 7. The raw discriminator scores of normal chest X-rays from both the training and test sets exhibit unimodal shapes that align well with the fitted Gaussian curves. After standardization using the mean *μ* and standard deviation *σ* calculated from 20% normal training samples, the standardized score of test normal samples also conforms to the standard normal distribution pattern. Normal *Q*–*Q* plots provide further supplementary validation: the vast majority of data points lie along the theoretical diagonal of normal distribution, with only trivial deviations at the extreme tails caused by borderline normal chest radiographs. The two groups of visual results collectively confirm that discriminator outputs of normal chest X-rays approximately follow a Gaussian distribution, which validates the rationality of the anomaly scoring scheme built upon Z-score and complements the previously missing distribution verification experiments.

#### 4.5.3 Visualization of reconstructed images

**Figure 8:**
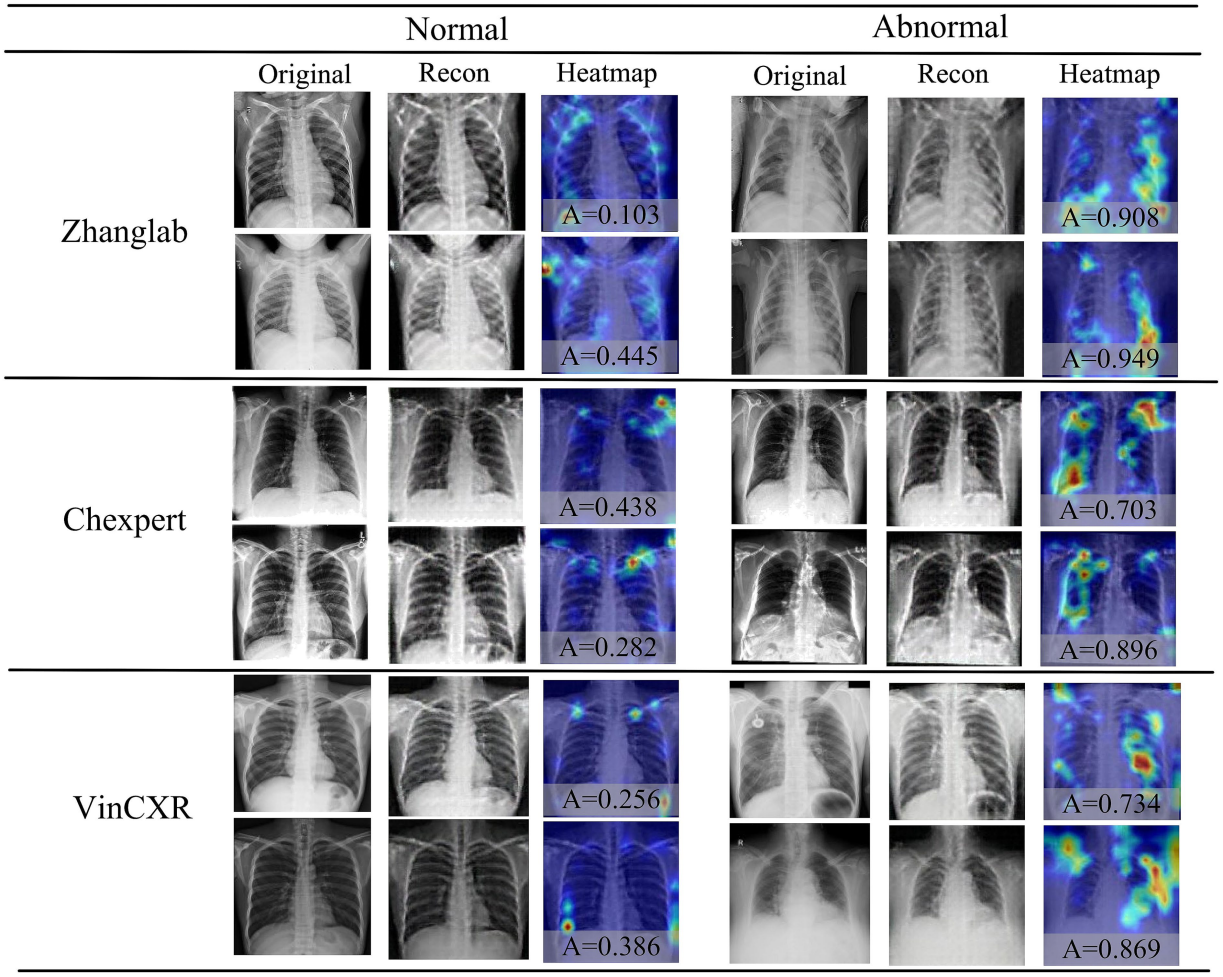
Reconstruction results and corresponding Grad-CAM heatmaps with anomaly score.

In Figure 8, we visualized DualA-AD, including original images (Original column), reconstructed images (Recon column) and heatmaps with anomaly score (Heatmap column) on all three datasets.

In the Recon column, the reconstruction results demonstrate DualA-AD’s capability to accurately reconstruct both normal and abnormal chest X-ray images while preserving fine anatomical details and overall structural integrity. This demonstrates the excellent ability of DualA-AD to capture the subtleties and overall structure of the image.

In the Heatmap column, Grad CAM (40) is employed to generate heatmaps of Dpixel which represents the probability of abnormal regions. Results show that heatmaps of normal images concentrate on edges or backgrounds, while heatmaps of abnormal images distinctly highlight diseased areas. Moreover, normal images have significantly lower anomaly scores than abnormal images. These indicate that DualA- AD effectively locates and quantifies abnormal regions, showing strong performance in chest X-ray anomaly detection.

### 4.6 Hyperparameter experiments

To determine the loss weight α for the single-image feature consistency in the FCM module, we conduct hyperparameter ablation experiments, as shown in Table 4. The FCM regularizes the feature distribution of normal samples collaboratively through dual constraints of single-image and cross-image feature consistency. Experimental results demonstrate that the model achieves the peak AUC of 91.94 when the loss weight is set to 0.05. This weight moderately suppresses intra-image feature drift within each chest X-ray without occupying the optimization space of the cross-image consistency branch for clustering global normal sample features, realizing the optimal coordination of the two constraints. Accordingly, we set 0.05 as the default weight for the single-image feature consistency loss throughout all experiments.

**Table 4:** Hyperparameter analysis of weight for single-image feature consistency loss in FCM.

α	AUC	ACC	F1
0.04	88.23	81.25	84.70
**0.05**	**91.94**	**86.46**	**89.11**
0.06	90.39	82.53	85.04
0.08	88.95	78.84	80.92

Bold marks best result.

### 4.7 Analysis of statistical and computational efficiency

#### 4.7.1 Analysis of statistical

To validate our experimental findings and assess their statistical significance, we quantified model classification confidence by calculating the distance from each sample to its corresponding decision threshold. Based on the distance gaps between our improved model and the SOTA model, paired *t*-tests and Wilcoxon signed-rank tests were implemented on three independent datasets (ZhangLab, CheXpert, and VinCXR). Table 5 shows that the *p*-values derived from both statistical tests are below the significance level of 0.05 across all datasets. This evidence indicates that despite the modest performance gain of our approach compared with the SOTA model, such improvement is statistically significant.

**Table 5:** The statistical analysis on three datasets.

Datasets	*T*-statistic	*T p*-value	Wilcoxon *p*-value
ZhangLab	6.3496	4.16 × 10^−10^	2.3 × 10^−11^
Chexpert	3.4177	0.00068	1.91 × 10^−4^
VinCXR	2.7515	0.00599	0.0154

#### 4.7.2 Analysis of computational efficiency

To comprehensively evaluate the performance of the discriminator, FCM and DAS components, comparative analysis is conducted across four core metrics in this experiment. Specifically, training time is defined as the average time cost of a single iteration over 300 training epochs; total parameters cover all trainable and frozen parameters of the model; peak VRAM records the maximum GPU memory consumption monitored throughout the entire training cycle; inference latency (Lat) is measured on the DAS module, representing the average single-sample inference time on the test set with a batch size of 1.

As shown in Table 6, the progressive integration of functional modules does not lead to a substantial expansion in parameter size. Even the full model equipped with all components maintains a total parameter volume below 120 MB. Although the introduction of the FCM module raises the per-epoch training time and peak VRAM usage, thereby increasing resource overhead during training, it exerts negligible impact on inference latency. This reveals that the supplementary modules only take effect in the training phase without impairing online inference efficiency. In summary, the proposed framework features lightweight parameter storage and high inference speed, which verifies its practical deployment feasibility under resource-constrained scenarios.

**Table 6:** Computational efficiency comparison of different module combinations.

Moudle	Train time (s)	Total parameters (MB)	Peak VRAM (MB)	Inference latency (ms)
MAE	–	111.91	–	–
MAE + $D_{\text{pixel}}$	21.2	112.81	2,294.01	14.19
MAE + DAL	24.94	113.28	3,014	14.05
MAE + DAL + FCM	524.34	116.79	5,151	14.15

### 4.8 Analysis of different disease categories

The Chexpert test set encompasses 12 categories of thoracic diseases, with a single chest radiograph potentially containing multiple abnormalities. We recorded the numbers of false positive and false negative samples from one experiment, as shown in Table 7. The table indicates that there were 250 normal and 250 abnormal samples in the test set; our method achieved a specificity of 77.60% and a recall of 76.0%. These two metrics are well-balanced and both at relatively high levels, with close values, demonstrating that the method achieves a good trade-off between reducing missed diagnoses and suppressing false alarms without obvious bias, thus exhibiting strong applicability in clinical screening scenarios.

**Table 7:** False positive and false negative data on the Chexpert dataset.

Normal	Abnormal	TP	FP	TN	FN	Specificity	Recall
250	250	190	56	194	60	77.60	76.0

Meanwhile, we counted the sample size and missed cases of each disease category on the Chexpert test set, as listed in Table 8. The proposed model achieves outstanding performance on clinically prevalent lesions with large-scale grayscale abnormalities, including pleural effusion, lung opacity, mild pleural abnormalities and edema, with the false negative rate of all categories controlled within 20%. This verifies that the model can stably extract large-range pathological texture features in the lung. Nevertheless, the model still has obvious limitations, as it struggles to identify fractures, pneumothorax and tiny focal lung lesions. Observations on original chest X-rays show that such abnormalities mostly present as thin cortical fracture lines, visceral pleural margins or focal nodules. Their visual features have negligible spatial-scale differences from normal tissue textures, which eventually leads to missed detections.

**Table 8:** Thoracic disease test statistics on the Chexpert dataset.

Disease categories	Test set sample size	False negative size	Percent
Fracture	21	8	0.38
Pneumothorax	22	7	0.32
Lung lesion	17	5	0.29
Consolidation	46	12	0.26
Cardiomegaly	46	10	0.22
Pneumonia	33	7	0.21
Atelectasis	73	15	0.21
Lung opacity	113	23	0.20
Enlarged cardiome diastinum	28	5	0.18
Pleural effusion	107	19	0.18
Edema	35	5	0.14
Pleural other	25	3	0.12

Overall, DualA-AD demonstrates a favorable sensitivity–specificity trade-off in unsupervised chest X-ray anomaly detection; however, its ability to discriminate subtle linear abnormalities and certain normal variations remains limited. Future research should consider introducing a local fine-grained feature extraction module that focuses on regions prone to minute lesions, such as the ribs, pleura, and lung margins, to strengthen the capture of subtle pathological features.

### 4.9 Generalization analysis

#### 4.9.1 Cross-dataset generalization evaluation

We design two comparative experiments to validate cross-dataset generalization. One model is trained on the ZhangLab dataset and tested on the Chexpert dataset, while the baseline model is trained and tested within the Chexpert dataset. As shown in Table 9, all evaluation metrics moderately decline under cross-dataset settings: AUC drops by 1.88%, accuracy decreases by 2.70%, and F1-score falls by 0.97%. Such performance degradation mainly results from domain shifts caused by inconsistent imaging equipment and divergent patient populations between the two datasets, which slightly impairs the model’s discriminative performance on unseen data distributions. Nevertheless, no dramatic performance collapse is observed, demonstrating that the proposed DualA-AD model achieves favorable cross-dataset generalization and can adapt to chest X-ray images from different sources. Based on the experimental results, training and test samples should be collected with identical imaging equipment whenever possible to mitigate performance interference caused by external imaging discrepancies.

**Table 9:** Cross-dataset validation.

Train dataset	Test dataset	AUC	ACC	F1
ZhangLab	Chexpert	81.25	74.8	75.95
Chexpert	Chexpert	83.13	77.50	76.92

#### 4.9.2 Noise robustness test

Given that the quantum noise of X-ray imaging follows Poisson statistics, reduced radiation doses lead to insufficient photon counts and intensified noise. Therefore, we inject Poisson noise into the ZhangLab dataset to simulate noise characteristics under low-dose conditions and verify the model’s noise robustness.

As shown in Table 10, the fluctuations of all key metrics are less than 1.2%, which far exceeds the 5% stability threshold commonly required in medical imaging. This demonstrates the strong anti-noise capability of the model. Although accuracy drops slightly by 1.1% and F1-score decreases by 1.2%, AUC remains nearly unchanged, indicating that the overall discriminative power of the model is inherently robust to quantum noise.

**Table 10:** Robustness experiments adding Poisson noise on the ZhangLab dataset.

Noise	AUC	ACC	F1
No	91.89	85.50	88.02
Poisson noise	91.94	86.46	89.11

## 5 Conclusion

In the paper, we propose DualA-AD for unsupervised anomaly detection in chest X- rays. In training, DualA-AD integrates spatial and frequency domains to capture detail and global information, improving normal distribution learning. Meanwhile, multi-layer feature differences within single image and similarity differences across multiple images improve the local and overall consistency between reconstructed and original images, effectively enchancing feature alignment. In testing, a new anomaly score is constructed by fitting the pixel discriminator’s output on the training set as the real distribution and quantifying the deviation of images to real distribution to enhance image distinguishability. DualA-AD outperforms the SOTA method in three datasets, demonstrating its potential for large-scale lung disease screening.

The DualA-AD unsupervised chest X-ray anomaly detection model developed in this study focuses on pre-screening for pulmonary diseases rather than fine-grained disease-specific diagnosis. In clinical settings, it is applicable to four major scenarios: large-scale infectious disease screening, routine preliminary screening in health checkups and outpatient clinics, consultations in primary healthcare institutions, and high-volume radiology report generation. The model can be deployed at the image acquisition end to assist technicians and junior physicians in rapid pre-screening. Clinical surveys (addressing the needs of physicians from outpatient/emergency departments, health checkup centers, and radiology departments) indicate that the core requirement in high-throughput clinical scenarios is rapid binary classification screening.

From a clinical safety perspective, multi-disease classification risks misdiagnosing rare lesions as common inflammation due to scarce training samples. Binary classification screening, by contrast, only flags normal and abnormal, prompting further investigations such as contrast-enhanced CT or biopsy, thereby effectively preventing missed rare disease diagnoses. This is precisely the rationale behind our use of unsupervised anomaly detection.

Although the DualA-AD model achieves efficient preliminary screening for clinically prevalent lesions, it still has inherent limitations. The model is deficient in extracting features of subtle linear lesions (e.g., fractures and pneumothorax) and intrapulmonary focal tiny lesions. Such lesions have texture features with low discriminability from normal anatomical tissues, which easily leads to missed diagnoses and results in deficiencies in the fine-grained identification of tiny lesions.

Future research will optimize and iterate the model in two main directions. First, a lightweight classification head will be appended to DualA-AD, and the model will be fine-tuned using a small amount of elaborately annotated medical image data. While retaining its original high sensitivity for abnormal lesion warning, an integrated inference system combining unsupervised anomaly screening and supervised disease classification will be constructed. Second, a local fine-grained feature extraction module will be introduced to perform targeted feature enhancement on high-incidence regions of tiny lesions including ribs, pleura and lung margins, to address the model’s weakness in characterizing subtle pathological features. Furthermore, we will cooperate with multiple tertiary hospitals to prospectively build an image dataset of rare thoracic diseases, improve the model’s generalization performance on long-tailed samples, and further advance its practical deployment in real clinical environments.

## Data Availability

The original contributions presented in the study are included in the article/supplementary material, further inquiries can be directed to the corresponding author.
